# Editorial: The Endocrine Regulation of Systemic Energy Homeostasis Under Physiological and Pathological Conditions

**DOI:** 10.3389/fphys.2020.602942

**Published:** 2020-10-29

**Authors:** Hui-Ying Lim, Weidong Wang, Yoshimi Nakagawa, Krystyna Pierzchala-Koziec

**Affiliations:** ^1^University of Oklahoma Health Sciences Center, Oklahoma City, OK, United States; ^2^Department of Research and Development, University of Toyama, Toyama, Japan; ^3^Department of Animal Physiology and Endocrinology, University of Agriculture, Krakow, Poland

**Keywords:** energy metabolism, insulin-like growth factors, cytokines, steroid hormones, receptor tyrosine kinase, bone remodeling, myostatin, diabetic peripheral neuropathy

The maintenance of systemic energy homeostasis is vital for organismal fitness and viability. This Research Topic is aimed at furthering our understanding of how systemic metabolic homeostasis can be achieved, by providing an overall view of how endocrine factors act, the receptors through which the endocrine factors elicit their effects, the complications arising from deranged energy metabolism, and finally the new evolutionary aspects of metabolic factors.

Numerous factors, including the cytokines and hormones, are known to play important roles in mediating tissue-tissue communications essential for achieving overall metabolic equilibrium. Steroid hormones, produced and secreted primarily by the adrenal cortex, play an important role in regulating energy metabolism and stress responses. An original research article by Ieka et al. reported for the first time the presence of enzymatic activities associated with steroidogenesis in the rat salivary glands, thereby indicating that steroid hormones are generated throughout the body and could work with other metabolic factors to mediate cross-talks between tissues or as metabolic regulators within specific tissues. Another important hormone of energy metabolism, ghrelin, is produced and released by the stomach and conveys information to the hypothalamus to enhance the use of carbohydrates and reduce fat utilization. Ghrelin also directly stimulates adipogenesis in mature rat adipocytes; however, its role in the preadipocytes is not clear. In an original research article, Miao et al. elucidated that ghrelin enhanced the proliferation of mouse 3T3 preadipocytes and human primary preadipocytes and suppressed their differentiation. These findings provide further understanding of the previously-unrecognized roles of ghrelin on adipocyte lipid metabolism. Cytokines are critical endocrine factors in systemic energy homeostasis regulation. In a review article, Shi et al. revealed deeper insights into the negative roles of various cytokines in the induction of metabolic dysfunction, which to this far are still not well-understood beyond their associations with lipid accumulation and inflammation in tissues. The notion of utilizing these cytokines as biomarkers for the early detection of metabolic disorders was also discussed in the review. Khan, in a review article, discussed the regulation of metabolic factors, emphasizing the mechanisms by which insulin-like growth factor (IGF)-binding protein-2 (IGFBP-2), a pleiotropic polypeptide, controls the bioavailability and localization of IGF-I and IGF-II in the central nervous system, and their functions on neuronal growth and development.

Through receptors, metabolic factors effectively elicit their effects in target organs to direct metabolic homeostasis. A review article by Zhao et al. focuses on the receptor tyrosine kinase (RTK) family of cell surface receptors, which respond to hormones, cytokines, and growth factors to trigger a diverse set of cellular and metabolic signaling pathways. The article describes the emerging molecular mechanisms of RTK signaling and highlights the unexpected roles for receptor families in regulating glucose and lipid metabolism. In another review article by Hardy and Fernandez-Patron, readers are introduced to the theme of bone remodeling by the extracellular matrix metalloproteinases (MMPs). Understanding bone remodeling, a process whereby bones renew themselves, is germane to our theme of systemic metabolic control and balance as the skeleton exerts an important endocrine regulation of glucose homeostasis and also bone cells regulated by certain hormones may send signals to influence the hormone-producing cells under a feedback loop. Building on the topic of glucose homeostasis, Yang et al. in a review article focuses on diabetic peripheral neuropathy (DPN), a common chronic complication of diabetes mellitus that arises from glucose metabolism dysregulation. With diabetes mellitus now reaching epidemic proportion, it is expected that the incidence of DPN will increase dramatically. This review describes recent advances in the diagnosis of DPN and the precision medicine techniques that may be used to improve treatment of this debilitating disease. Last but not least, an original research article by Segev-Hadar et al. reported the identification of a second myostatin gene (*mstn2*) in the aquaculture fish species, Nile tilapia. Myostatin is a growth and differentiation factor that regulates skeletal muscle development which in turn impinges on the production of myokines essential for systemic energy metabolism. Results from this article showed that *mstn2* is not expressed in muscle cells but in the brain, and that centrally- and muscle-expressed *mstn* genes differ in their responsiveness to diverse challenges, suggesting a differential gene- and tissue-specific regulation of their expression.

In summary, this Research Topic provides an integrated overview of the endocrine regulation of systemic metabolic homeostasis and brings forward our understanding of this complex but important field.

## Author Contributions

H-YL wrote the editorial. WW, YN, and KP-K critically reviewed the editorial. All authors contributed to the article and approved the submitted version.

## Conflict of Interest

The authors declare that the research was conducted in the absence of any commercial or financial relationships that could be construed as a potential conflict of interest.

